# Post-stroke diabetes management: a qualitative study

**DOI:** 10.3389/fneur.2024.1364217

**Published:** 2024-04-12

**Authors:** Jonathan Hewitt, Hala F. Azhari, Martin O’Neill, Alexander Smith, Terence Quinn, Jesse Dawson

**Affiliations:** ^1^School of Geriatric Medicine, Cardiff University, Cardiff, United Kingdom; ^2^College of Medicine and Pharmacy, Umm Al-Qura University, Makkah, Saudi Arabia; ^3^Social and Economic Research Data and Methods, Cardiff University, Cardiff, United Kingdom; ^4^Clinical Research and Innovation Centre, St Woolos Hopsital, Newport, United Kingdom; ^5^School of Cardiovascular and Metabolic Health, University of Glasgow, Glasgow, United Kingdom

**Keywords:** diabetes, stroke, stroke survivors, healthcare professionals, focus groups, qualitative study

## Abstract

**Introduction:**

Diabetes is associated with an increased risk of stroke. In many cases, a diabetes diagnosis may predate a stroke; however, diabetes is often diagnosed during the hospital admission following a stroke. To explore the experiences of stroke survivors as they cope with a new diabetes diagnosis, particularly regarding developing an effective strategy for managing the disease.

**Methods:**

A qualitative grounded theory approach was used that employed focus group interviews with participants, including clinicians and stroke survivors, to develop a holistic understanding of primary and secondary stroke care services and the experiences of those accessing them.

**Results:**

Clinicians believed they were not optimally equipped to manage diabetes as a condition. They believed more emphasis should be placed on self-management, which would be better managed by lifestyle changes than medication alone. Conversely, stroke survivors with diabetes experienced an additional burden associated with the diagnoses but relied on clinicians to manage their diabetes and believed the clinicians were failing if they were unwilling or unable to achieve this.

**Discussion:**

The research highlights the tensions between stroke survivors and healthcare professionals. Stroke survivors relied on the healthcare teams to provide the optimal treatment when they had recently undergone a significant health event where they had experienced a stroke and received a diabetes diagnosis. However, the healthcare teams, while recognizing the importance of a holistic and comprehensive treatment package, struggled to provide it due to resource limitations. To optimize post-stroke diabetes self-management education, a strategic framework that prioritizes patient empowerment and interdisciplinary collaboration is paramount. Tailoring educational interventions to align with individual patient profiles—considering their unique health status, personal preferences, and cultural context—is essential for fostering self-efficacy. Such a strategy not only empowers patients to take an active role in managing their diabetes post-stroke but also contributes to superior health outcomes and an elevated standard of living.

## 1 Introduction

Stroke is a major cause of morbidity and mortality, accounting for 11% of all deaths worldwide (1). The link between diabetes and stroke is well-established, with the incidence of diabetes more than doubling the risk of stroke for an individual (2), and it is a contributing factor to 20% of strokes in the United Kingdom (UK) (3). Diabetes plays a critical role in both the incidence and outcome of stroke, acting as a significant factor in the pathophysiology and clinical management of stroke events. The presence of diabetes is known to worsen the prognosis following an acute stroke, with various biological factors contributing to poorer recovery profiles in diabetic patients (4). These factors include, but are not limited to, heightened inflammatory responses, impaired blood flow, and increased oxidative stress. The management of diabetes in post-stroke settings is thus of paramount importance, as it involves not only the control of blood glucose levels but also the careful monitoring of these biological processes. Addressing diabetes effectively in stroke patients is crucial for improving their recovery outcomes and reducing the risk of subsequent strokes.

Almost a third of stroke survivors first become aware of their diabetes after admission to hospital following a stroke (5). Therefore, the diagnoses and aftercare for stroke and diabetes are strongly linked. A key element of the treatment of stroke and related diabetes following hospital admission aims to prevent further cerebrovascular events. An intrinsic element of this treatment is implementing drug therapies to lower and control blood sugar, cholesterol, and blood pressure (6). However, implementing these drug therapies is mediated by several barriers, such as the stroke survivor’s willingness to cooperate and comply with and adhere to such a therapy regime.

Multiple barriers to medication compliance have been identified and characterized as specific to the individual, medication, or healthcare setting post-stroke. Previous studies (7) identified that knowledge of stroke prevention therapies and medication compliance is suboptimal among stroke survivors. They found several competing factors influencing both the treatment prescribed and offered by the physician and the stroke survivors’ reception of or compliance with that treatment. These factors concerned issues such as knowledge of stroke and diabetes severity and etiology, medication efficacy and associated side effects, and inadequate provision of information (8). Regardless of whether adherence to cardiovascular therapies influenced the risk of further cardiovascular disease (CVD), a significant proportion of survivors with stroke did not adhere to their medications, and as much as 9% of all CVD events in Europe could be attributed to poor adherence to cardiovascular medications alone in people with or without diabetes (7).

Further evidence concerning factors affecting stroke survivors’ adherence to treatment relates to the poor uptake of these medications (8). Therefore, this qualitative research aims to explore the attitudes and contributing factors impacting initially deciding on a particular diabetes treatment strategy and adhering to it following a stroke.

## 2 Methods

The study protocol was registered using the UK Integrated Research Application System, (reference number: 238470) with an ethical approval was obtained from the London – Harrow Research Ethics Committee (reference number: 17/LO/2122) (9). The study was conducted according to the principles expressed in the Declaration of Helsinki.

### 2.1 Study design and participants

The study adopted a qualitative “*grounded theory*” (10) approach and used focus groups with stroke survivors diagnosed with diabetes before or following their stroke and healthcare professionals directly involved in the care of stroke survivors with diabetes.

Focus group interviews were conducted with the study participants on two sites, one in the South Wales and the other in the Scotland health board centers. The participant selection criteria and recruitment process were as follows: stroke survivors with a history of diabetes were identified through the clinical care teams in the hospitals at both sites. The healthcare professionals were approached through special interest groups of their professional networks. These healthcare professionals all specialized and were directly involved in caring for people with stroke and/or diabetes. Purposive sampling was undertaken to achieve a maximum spread of age, socioeconomic status, sex, and disability. A minimum of two individuals from each sex were surveyed (geriatric and stroke consultants, diabetes specialists, occupational therapists, general practitioners, and clinical stroke nurses). The final number of interviews was determined by the point of data saturation.

Stroke survivors and healthcare professionals participated in separate focus groups, received an information sheet, and provided written informed consent to participate in this study. All participant data were anonymized. No any participant identifying of information in this study.

### 2.2 Aim of the study

This study explored the factors influencing the diabetes treatments prescribed and offered by physicians and the reception of and compliance with that treatment by stroke survivors. The research with the stroke survivors explored their views on the links between diabetes and stroke and between diet and diabetes and how they viewed different treatment approaches. For the healthcare professionals, the focus groups explored the issues impacting stroke survivors’ care and treatment post-stroke diabetes management.

### 2.3 Format of the focus groups

To facilitate the focus group meetings, the focus group interviews were conducted by researchers experienced in qualitative methodology. Focus groups were conducted in neutral territory away from the study participants’ homes or the hospital in what aimed to provide a “*safe space*” for exploring the topics covered. The focus groups were structured to enable in-depth investigation of the participant’s personal perspectives, using an open-ended line of questioning. A topic schedule guided the line of questioning and prompted further discussion. Discussion topics included attitudes to secondary prevention care, medication beliefs, adherence to treatment, and barriers to uptake. All interviews were digitally recorded during the focus groups and transcribed directly in preparation for coding as there were no preparatory training sessions were conducted.

### 2.4 Data analysis

An iterative process of data collection and data analysis was undertaken. Transcripts were initially read by one of the researchers involved in conducting the focus groups to resolve any inaccuracies occurring through the transcription process by listening to the recordings, ensuring the reliability of interpretation. The interviews were then transcribed and coded to manage and code the data. The transcripts were analyzed adopting a grounded theory method approach, which was followed by a constant comparative analysis approach (10), in which key points were identified from the data and coded individually. Initially, chunks of data were coded. Codes were then grouped into similar concepts and themes, and categories were formed. A process of identification and refinement of categories followed. As groups were compared further, more abstract categories were developed until the core themes emerged. Inconsistencies were resolved through discussion with a third experienced researcher until a consensus was reached on the final themes.

We followed the Reporting of Qualitative Research checklist criteria (11) in the reporting of this study.

## 3 Results

Three focus groups were completed from January 2019 to July 2022: two with multidisciplinary healthcare professionals and one with stroke survivors. In total, sixteen stroke survivors and thirty-two healthcare professionals consented to participate in the focus groups. Stroke survivors’ participants were recruited from Glasgow healthcare hospital (eight men and eight women) aged 54–80 years, 23 (48%) were women, who had diabetes and recovered from a stroke. Healthcare professionals’ participants were recruited from two health board centers in Scotland and Wales (four stroke consultants and geriatric, two diabetes specialists, two general practitioners, three occupational therapist, two stroke trainee doctors, and three clinical stroke nurses). The study participants’ baseline characteristics are detailed in Table 1.

**Table 1:** Baseline characteristics of the participants included in the qualitative study.

Focus group	Participants’ background			
	No.	Age	Sex	Baseline characteristics
Focus group I (Glasgow) Stroke survivors	1	55	Female	Raised blood pressure, diabetes mellitus, ischemic stroke
	2	80	Male	Diabetes mellitus, raised blood pressure, ischemic stroke
	3	65	Male	Raised blood pressure, diabetes mellitus, ischemic stroke
	4	65	Male	Raised blood pressure, diabetes mellitus, ischemic stroke
	5	66	Female	Diabetes mellitus, raised blood pressure, ischemic stroke
	6	54	Female	Diabetes mellitus, ischemic stroke, raised blood pressure
	7	65	Male	Ischemic stroke, diabetes mellitus, raised blood pressure
	8	61	Female	Ischemic stroke, raised blood pressure, diabetes mellitus
	9	62	Male	Ischemic stroke, diabetes mellitus
	10	68	Female	Ischemic stroke, diabetes mellitus, raised blood pressure
	11	55	Male	Ischemic stroke, diabetes mellitus
	12	70	Female	Raised blood pressure, ischemic stroke, diabetes mellitus
	13	79	Male	Ischemic stroke, diabetes mellitus
	14	69	Female	Ischemic stroke, diabetes mellitus, raised blood pressure
	15	65	Male	Ischemic stroke, diabetes mellitus
	16	50	Female	Ischemic stroke, diabetes mellitus
Focus group II (Glasgow National Health Service) Healthcare professionals	1	50	Female	Consultant geriatrician/stroke
	2	55	Male	Consultant geriatrician
	3	46	Male	Consultant/stroke
	4	45	Female	Consultant/stroke
	5	55	Male	Occupational therapist
	6	49	Male	Occupational therapist
	7	52	Female	Occupational therapist
	8	56	Male	Diabetes specialist
	9	60	Female	Endocrinologist
	10	39	Female	Trainee doctor/stroke
	11	32	Male	Trainee doctor/stroke
	12	40	Male	Nurse/stroke
	13	42	Female	Nurse/stroke
	14	39	Female	Clinical stroke nurse
	15	35	Female	General practitioner
	16	32	Male	General practitioner
Focus group III (Wales National Health Service) Healthcare professionals	1	50	Female	Consultant geriatrician/stroke
	2	56	Male	Consultant/stroke
	3	60	Female	Consultant/stroke
	4	59	Male	Consultant geriatrician/stroke
	5	44	Male	Occupational therapist
	6	46	Male	Physiotherapist
	7	51	Female	Occupational therapist
	8	38	Male	Endocrinologist
	9	59	Female	Diabetes consultant
	10	39	Female	Trainee doctor/stroke
	11	35	Male	Trainee doctor/stroke
	12	30	Female	General practitioner
	13	33	Male	General practitioner
	14	35	Male	Nurse/stroke
	15	40	Male	Clinical stroke nurse
	16	46	Female	Nurse/stroke

The emerging themes concerned how seriously stroke survivors took their treatment and lifestyle choices, their knowledge of stroke and diabetes medication, and possible side effects.

### 3.1 Stroke survivors focus group

The stroke survivors appeared to believe that they were given no clear direction or advice that the stroke and diabetes were linked. The only connection they had made between the two was that they would be prescribed an antidiabetic medication following the diagnosis. As a stroke survivor stated, “*it stopped at that*,” which did not appear to indicate that they had made a connection with any lifestyle change other than taking the medication:

“Yeah, I discovered I had type 2 diabetes as a result of my heart attack when they tested for it, and they said, ‘Oh, and you have got type 2 diabetes.’ And it sorts of stopped at that, apart from they said, ‘Oh, here you are, take gliclazide because that helps.’”

“No, I knew that with diabetes it’s – your sugar has to be controlled after it’s discovered, but I do not know controlling the sugar will prevent diabetes; it’s more a sort of this is the way to deal with it, to cut down your sugar. But I did not know I had it, so I’ve not had a thought of watching sugar other than just my normal weight. I’ve put on weight since my stroke, but it was just part of just being normal. I’ve never stopped, ‘I’m enjoying that. I’ll maybe have another one before I go,’ but never – that’s going to lead to diabetes; that’s why people who have diabetes do not eat them.”

“I mean it takes so long just to deal with the shock of the life-changing event that’s happened to you, so everything else has to be over and above. It’s hard enough because of it.”

Although stroke survivors were very concerned about their diabetes and keen to manage it to avoid further health-related issues, they were unsure whether they were managing it optimally and welcomed further advice and guidance. Stroke survivors also expressed concern about the possible side effects of drugs and their potential impact on their health:

“If somebody said to me, take this pill and you will not have a stroke, I’d bite their arm off to go after that, and it’s as simple as that because I’m looking round here and I’m one of the least affected by the stroke that I had. I do not want another one, and I’m now concerned that diabetes is sneaking up on me and I do not know how to cure it. What I’ve done is I’ve stopped the sugar. I’ve not had sweeties. We do not have cakes and biscuits in our house. We do all these things because my wife’s type 2 as well, and you do all these things; if somebody says, ‘Here’s a way of sorting it,’ for heaven’s sake, let me know now because I do not want to have my leg off and then somebody says, ‘Oh, you should have been taking that pill.’ I want to know now.”

Although the stroke survivors were concerned about the impact the stroke and diabetes had and continued to have on their health, they were unsure or uninformed regarding the best course of action they could follow to mitigate their position. Although they appeared to rely on the physician for advice, guidance, and medication to cure their condition, the interviews provided no clear indication that they believed they were receiving these:

“what’s the point? Really, it’s – you’ll maybe get the balance. If I take this, that and this, what will I get away with? You know, how will I be able to cope, or if I do not take this, where am I heading? And it’s just a question of balance and you are relying on the person that’s talking to you, the doctor, to work out that balance, take this, take this, take this and this goes down. But other than that, you guess you’ll be the perfect weight, the perfect, but you might have a stroke.”

### 3.2 Healthcare professionals focus groups

Healthcare professionals explored issues they believed directly impacted their treatment decisions for stroke survivors diagnosed with diabetes. Their general resource issues within the healthcare system included the need to access staff specializing in diabetes:

“In our [GP] practice the challenge is getting enough diabetic nurses. We’re working hard to try and train the diabetic team, because they quite often do the blood tests and then just bring them back three months later and not actually changing management in any way. So, trying to intensify our treatment to make sure the nHbA1c is actually treating the target. Getting patients to comply. Numbers, vast volumes of numbers I think – this year alone, we seem to be picking up more and more pre-diabetics. Even the people that do not actually look like they would be at risk of pre-diabetes. And then you have got this whole cohort of patients that need education and monitoring and working through. So, I think it’s, yeah, just volumes of numbers and getting the nurses to be trained up in order to deal with that.” (Primary care physician, Wales).

Issues impacting the provision of support and guidance for stroke survivors are highlighted in the earlier interviews with the stroke survivors. Resource issues, particularly regarding staff and training and the increasing demand burden, directly impact on the service they can provide. These issues, particularly increasing demand, impact providing a basic service without being able to address complementary follow-up services, such as monitoring compliance and providing education and support. This resource issue was further compounded by the recognition of a need for additional pastoral and emotional support for those recently diagnosed with stroke and diabetes:

“I’ve not seen an awful – I do not think I’ve seen an awful lot of newly diagnosed, but obviously they are the ones who suddenly need … a lot of chat … about stroke and diabetes and diet and managing it, but the ones I – the few I can think of, yes, just it’s a lot for them to take on and just giving them – finding them information about it all.” (Diabetes specialist nurse, Scotland).

The healthcare professionals recognized the importance of pastoral and emotional support for those newly diagnosed but also recognized that providing such a service is particularly resource intensive. These competing demands between what was believed to be required by stroke survivors and what, due to resource implications, was possible to provide were also mirrored in the competing aims of medications. A key area of discussion among the healthcare professionals was the competing pressures impacting their prescribing for stroke survivors with diabetes. Pressure to comply with recommended guidelines also featured as a concern for healthcare professionals:

“I think if there’s robust evidence and a guideline that comes out that’s clear, I mean GPs are used to having to adjust their practice and moving with the times, and I do not think – I think it would just need to be clear. What becomes problematic is if the guideline is like the current one, where you cannot follow it and the evidence … – you know, it’s a bit wish-washy. There’s evidence for lots of different things. So, I think if there’s a clear guidance then GPs would be fine with it because things come out all the time, you have to change your practice.” (Secondary care physician, Scotland).

## 4 Discussion

As this paper has shown, several factors impact healthcare professionals and stroke survivors regarding managing post-stroke diabetes diagnoses. These factors can be viewed as several interacting themes. First, stroke survivors’ knowledge/lack of knowledge concerned the etiology and treatment of diabetes, particularly regarding diet as causing diabetes rather than simply requiring management after diagnosis. Second, limited resources for the treatment team impacted the level of service they could provide despite a recognition that advice and support could be particularly valuable in increasing compliance and the overall management of the condition. Third, uncertainty existed for stroke survivors regarding how they could best manage their condition and for healthcare professionals regarding their concerns about polypharmacy, the uncertainty of drug interactions, and the implications for stroke survivors’ welfare. Finally, post-diagnosis treatment of diabetes and how this might impact further strokes were an issue, particularly concerning the role of the stroke survivors in monitoring their condition and medication while complying with recommended guidance.

The overriding themes and points of contradiction between stroke survivors and healthcare professionals were the level of information provided to stroke survivors and the degree to which expectations differed regarding who was responsible for managing post-stroke diabetes. Stroke survivors viewed this role as belonging to the healthcare professional; conversely, healthcare professionals considered post-stroke diabetes management the responsibility of the stroke survivor, especially regarding self-management.

Self-directed management of diabetes is a commonly accepted method of engaging (12) and empowering (13) stroke survivors to manage their condition. This is at least partially due to the chronic nature of diabetes and stroke and the need for treatments to be adhered to in a cost-effective community-based manner. This can only be achieved on a scale through the people with diabetes taking charge (14). The situation may differ slightly for people with diabetes who have suffered a stroke, which may explain the discrepancies highlighted in our study. Firstly, diabetes may be diagnosed at the same time as a stroke. In this situation, an individual is dealing with a stroke diagnosis and may be too overloaded with new and distressing diagnoses to comprehend and successfully self-manage diabetes in an acute situation. Additionally, cognitive impairment, which commonly accompanies a stroke, may further hamper a stroke survivor’s ability to correctly self-manage diabetes.

The actual situation may lie between these extremes, with healthcare professionals and stroke survivors showing an increased willingness to manage diabetes after a stroke. For example, in a large sample of stroke survivors in Wales, the frequencies of diabetes testing and the level of diabetes control achieved improved in the year after a stroke (5). This implies that not only were healthcare professionals engaged and tested diabetes more often, but also stroke survivors with diabetes had better diabetic control, a process achievable only if the person with diabetes also engaged.

In the context of existing literature based on the findings from the barriers to effective post-stroke comorbidity management in stroke survivors with diabetes COMPOSEd study (15) as a future strategy to ensure effective post-stroke diabetes management suggests incorporating pioglitazone into treatment protocols, given its potential benefits in improving insulin sensitivity and reducing cardiovascular risk factors in stroke survivors with diabetes. However, the study also highlights the need to address barriers such as medication adherence, comorbidity management, and healthcare access to optimize outcomes in this vulnerable population. Developing personalized care plans tailored to the unique needs of stroke survivors with diabetes, along with multidisciplinary collaborations involving healthcare providers, caregivers, and community resources, could enhance the delivery of comprehensive care and improve long-term prognosis.

## 5 Implications for future research

This study highlights that despite recognition by stroke survivors and healthcare professionals that a holistic and comprehensive regimen for managing a post-stroke diabetes diagnosis would be welcome, current resources cannot provide a comprehensive service. This has significant implications for service provision since, although healthcare professionals are fully aware of the importance of continued support, advice, and guidance in managing diabetes, they cannot provide it within the current service environment. Adopting such an approach could contribute to alleviating their concerns regarding the adverse impact of polypharmacy on stroke survivors since improving the management of the condition through diet and lifestyle changes should reduce the reliance on medication.

An effective strategy to empower education on self-management post-stroke for stroke survivors with diabetes involves a patient-centered approach that emphasizes empowerment and collaborative care. Group education programs should be designed to foster a sense of autonomy and self-determination, enabling patients to make informed decisions about their health and daily management of their condition. This includes providing tailored information that aligns with the stroke survivors’ values, lifestyle, and cultural background. Additionally, integration of advanced digital platforms and telemedicine health services can significantly enhance the accessibility and personalization of educational content, thereby ensuring continuity of care, facilitating sustained patient engagement, and self-care competencies (16). By focusing on empowerment and ongoing support, healthcare providers can help patients develop the skills and confidence needed to effectively manage their diabetes after a stroke, leading to improved health outcomes and quality of life.

The study adopted several steps to understand the benefits of self-management post-stroke diabetes for clinical practice. Adopting post-stroke diabetes self-management educational approach could contribute to alleviating the stroke survivors’ concerns regarding the adverse impact of polypharmacy post-stroke diabetes and would reduce their reliance on medication. Allowing flexibility in developing guidelines and instituting appropriate health education using input from the perspective of both healthcare professionals’ and stroke survivors would provide appropriate suggestions to incorporate into post-stroke diabetes self-management. Additional studies are needed to pinpoint stroke survivors who would find the service provision, rather than solely relying on medication, acceptable, while yielding the highest preventive benefits for before and after a stroke diabetes self-management.

## 6 Strength and limitations

As a theoretical framework for acceptability (17), this study combined the insights of healthcare professionals and stroke survivors to explore the applicability of service provision in the guidance for stroke survivors. Although we ensured equal opportunities for the participants to respond to our focus group interview questions, our study may be subject to a dominant respondent bias, where some participants may have continuously dominated the talk and influenced the opinions of the other respondents. The healthcare professionals were recruited from the primary and secondary Wales and Glasgow stroke centers to increase the probability of experience diversity. These experiences may exhibit variations across different geographic regions and within diverse patient demographics. However, social desirability bias may exist, where the professionals might answer as they consider acceptable rather than based on their actual practice in clinical settings. Although stroke survivors with diabetes were recruited from a single hospital in Glasgow, with such heterogeneity, some participants may not have felt fully able to articulate them as their opinions may have found unsupported by others. Another potential limitation was the small sample size of the qualitative study; however, a theoretical saturation was assured (18) and may still indicate a pattern of clinical practices that could inform a design for future clinical study.

## 7 Conclusion

This study suggested exploring methods to manage post-stroke diabetes from the perspectives of healthcare professionals and stroke survivors. Typically, healthcare professionals and stroke survivors identified a need to address the barriers to effective pre-and post-stroke diabetes management. The provision of services by healthcare professionals to guide the stroke survivors’ self-directed management could advance care in treating stroke survivors with post-stroke diabetes, allow greater treatment adherence, and increase the confidence of healthcare professionals in their practice. Further research is required to identify stroke survivors for whom service provision, as an alternative approach to pharmacological intervention alone, might be acceptable with the greatest pre-and post-stroke diabetes preventive benefits. The findings of this study inform the future of post-stroke trial design.

## Data availability statement

The original contributions presented in the study are included in the article/supplementary material, further inquiries can be directed to the corresponding author.

## Ethics statement

The study protocol was registered using the UK Integrated Research Application System (reference number: 238470) with an ethical approval was obtained from the London – Harrow Research Ethics Committee (reference number: 17/LO/2122) (NHS Health Research Authority, 2018). The study was conducted according to the principles expressed in the Declaration of Helsinki. The studies were conducted in accordance with the local legislation and institutional requirements. The participants provided their written informed consent to participate in this study. Written informed consent was obtained from the patients/participants for the publication of any potentially identifiable data included in this article.

## Author contributions

JH: Writing – review & editing, Writing – original draft, Project administration, Funding acquisition, Formal analysis, Data curation. HA: Writing – review & editing, Writing – original draft, Visualization, Validation, Software, Resources, Project administration, Methodology, Investigation, Funding acquisition, Formal analysis, Data curation, Conceptualization. MO’N: Writing – review & editing, Writing – original draft, Project administration, Methodology, Funding acquisition, Data curation. AS: Writing – review & editing, Writing – original draft, Supervision, Formal analysis, Data curation. TQ: Writing – review & editing, Writing – original draft, Supervision, Data curation. JD: Writing – review & editing, Writing – original draft, Supervision, Project administration, Data curation.

## References

[1] GBD 2019 Stroke Collaborators. Global, regional, and national burden of stroke and its risk factors, 1990–2019: a systematic analysis for the global burden of disease study 2019. Lancet Neurol. (2021) 20:795–820. doi: 10.1016/S1474-4422(21)00252-0 34487721 PMC8443449

[2] Lozano R Naghavi M Foreman K Lim S Shibuya K Aboyans V. Global and regional mortality from 235 causes of death for 20 age groups in 1990; and 2010: a systematic analysis for the global burden of disease study 2010. Lancet. (2012) 380:2095–128. doi: 10.1016/S0140-6736(12)61728-0, PMID: 23245604 PMC10790329

[3] Stroke Association. (2017). State of the nation: stroke statistics 2017. Available at: https://www.stroke.org.uk/sites/default/files/state_of_the_nation_2017_final_1.pdf(Accessed November 30, 2023)

[4] Bradley SA Spring KJ Beran RG Chatzis D Killingsworth MK Bhaskar SMM. Role of diabetes in stroke: Recent advances in pathophysiology and clinical management. Diabetes Metab Res Rev. (2021) 38:e3495. doi: 10.1002/dmrr.3495 34530485

[5] Robson R Lacey AS Luzio SD Van Woerden H Heaven ML Wani M. HbA1c measurement and relationship to incident stroke. Diabetic Med. (2016) 33:459–62. doi: 10.1111/dme.13057, PMID: 26683404 PMC5066734

[6] The PROGRESS Collaborative Group. Effects of a perindopril-based blood pressure lowering regimen on cardiac outcomes among patients with cerebrovascular disease. Eur Heart J. (2003) 24:475–84. doi: 10.1016/S0195-668X(02)00804-7 12633548

[7] Chowdhury R Khan H Heydon E Shroufi A Fahimi S Moore C. Adherence to cardiovascular therapy: a meta-analysis of prevalence and clinical consequences. Eur Heart J. (2013) 34:2940–8. doi: 10.1093/eurheartj/eht295, PMID: 23907142

[8] Kronish IM Diefenbach MA Edmondson DE Phillips LA Fei K Horowitz CR. Key barriers to medication adherence in survivors of strokes and transient ischemic attacks. J Gen Intern Med. (2013) 28:675–82. doi: 10.1007/s11606-012-2308-x, PMID: 23288379 PMC3631079

[9] NHS Health Research Authority. (2018). COMPOSEd – Barriers to Effective Co-morbidity Management Post-Stroke. Available at: https://www.hra.nhs.uk/planning-and-improving-research/application-summaries/research-summaries/composed-barriers-to-effective-co-morbidity-management-post-stroke/ (Accessed November 30, 2023)

[10] Glaser B. Strauss A. (1999). The discovery of grounded theory: Strategies for qualitative research. 1st Edn. New York. 282, e9780203793206. Available at: https://www.taylorfrancis.com/books/mono/10.4324/9780203793206/discovery-grounded-theory-barney-glaser-anselm-strauss

[11] Tong A Sainsbury P Craig J. Consolidated criteria for reporting qualitative research (COREQ): a 32-item checklist for interviews and focus groups. Int J Qual Health Care. (2007) 19:349–57. doi: 10.1093/intqhc/mzm042, PMID: 17872937

[12] Franek J. Self-management support interventions for persons with chronic disease: an evidence-based analysis. Ontario Health Technol Asses Series. (2013) 13:1–60. PMID: 24194800 PMC3814807

[13] Galdas P Fell J Bower P Kidd L Blickem C McPherson K. The effectiveness of self-management support interventions for men with long-term conditions: a systematic review and meta-analysis. BMJ Open. (2015) 5:e006620. doi: 10.1136/bmjopen-2014-006620, PMID: 25795688 PMC4368927

[14] Deakin TA Cade JE Williams R Greenwood DC. Structured patient education: the diabetes X-PERT Programme makes a difference. Diabetic Med. (2006) 23:944–54. doi: 10.1111/j.1464-5491.2006.01906.x, PMID: 16922700

[15] Azhari H Hewitt J Smith A O'Neill M Quinn T Dawson J. Pioglitazone and barriers to effective post-stroke comorbidity management in stroke survivors with diabetes. Neurosci J. (2024) 29:44–50. doi: 10.17712/nsj.2024.1.20230043 PMC10827012 38195138

[16] Tang TS Funnell MM Anderson RM. Group education strategies for diabetes self-management. Diabetes Spectr. (2006) 19:99–105. doi: 10.2337/diaspect.19.2.99

[17] Sekhon M Cartwright M Francis JJ. Acceptability of healthcare interventions: an overview of reviews and development of a theoretical framework. BMC Health Serv Res. (2017) 17:88. doi: 10.1186/s12913-017-2031-8, PMID: 28126032 PMC5267473

[18] Hennink M Kaiser BN. Sample sizes for saturation in qualitative research: a systematic review of empirical tests. Soc Sci Med. (2022) 292:114523. 34785096 10.1016/j.socscimed.2021.114523

